# LINC01420 RNA structure and influence on cell physiology

**DOI:** 10.1186/s12864-019-5538-z

**Published:** 2019-05-08

**Authors:** Daria O. Konina, Alexandra Yu. Filatova, Mikhail Yu. Skoblov

**Affiliations:** 1 Department of Biological and Medical Physics, Moscow Institute of Physics and Technology (State University), Dolgoprudny, 141701 Russian Federation; 2 Research Centre for Medical Genetics, Moscow, Russian Federation 115522; 3 Far Eastern Federal University, Vladivostok, 690090 Russian Federation

**Keywords:** LINC01420, NBDY, Long noncoding RNA, lncRNA, Cell migration, smORF, RACE, MTT, Wound healing

## Abstract

**Background:**

It was shown that the major part of human genome is transcribed and produces a large number of long noncoding RNAs (lncRNAs). Today there are many evidences that lncRNAs play important role in the regulation of gene expression during different cellular processes. Moreover, lncRNAs are involved in the development of various human diseases. However, the function of the major part of annotated transcripts is currently unknown, whereas different lncRNAs annotations tend to have low overlap. Recent studies revealed that some lncRNAs have small open reading frames (smORFs), that produce the functional microproteins. However, the question whether the function of such genes is determined by microprotein or RNA itself or both remains open. Thus, the study of new lncRNA genes is important to understanding the functional role of such a heterogeneous class of genes.

**Results:**

In the present study, we used reverse transcription PCR and rapid amplification of cDNA ends (RACE) analysis to determine the structure of the *LINC01420* transcript. We revealed that *LINC01420* has two isoforms that differ in length of the last exon and are localized predominantly in the cytoplasm. We showed that expression of the short isoform is much higher than the long. Besides, MTT and wound-healing assays revealed that *LINC01420* inhibited cell migration in human melanoma cell line A375, but does not influence on cell viability.

**Conclusion:**

During our work, D’Lima et al. found smORF in the first exon of the *LINC01420* gene. This smORF produces functional microprotein named non-annotated P-body dissociating polypeptide (NoBody). However, our results provide new facts about *LINC01420* transcript and its function.

## Background

After the appearance of the GENCODE project [1], the mRNA-centric paradigm for transcript annotation has dramatically changed. It was shown that the major part of the human genome (~ 62–75%) is transcribed and produces a large number of long noncoding RNAs (lncRNAs) [2, 3]. LncRNAs are transcripts with the length more than 200 nucleotides that do not possess long open reading frames (ORFs) [4]. They are actively studied relatively recently, and today there is not even a good understanding of their number. According to the current GENCODE release (version 29), 16′066 lncRNA genes were annotated in the human genome. At the same time, the FANTOM CAT project revealed 27′919 human lncRNA genes and predicted the potential functionality of ~ 69% of them [5].

Despite this, only a small number of lncRNAs has experimentally defined function [6]. It is already known, that they are involved in various gene-expression regulation processes on transcriptional [7–13] and post-transcriptional [14–16] levels. Besides, lncRNAs play a role in the development of various human diseases [17]. The lncRNADisease database contains entries about 914 lncRNAs associated with 329 diseases [18], including different cancer types, genomic imprinting disorders, neurodegenerative, cardiovascular diseases and other pathologies. In some cases, non-coding transcripts play a key role in the molecular pathogenesis of the diseases. Thus, lncRNAs have the potential to be used as biomarkers and therapeutic targets [19].

There are several lncRNA annotations, which tend to have low overlap. For example, FANTOM CAT and GENCODE have 25,7% and 57,8% common genes respectively. Moreover, the most annotations of transcripts are 5′-end and 3′-end incomplete [20]. The challenge of lncRNA gene annotation is related to their differences from mRNAs, such as weak conservation, low and tissue-specific expression, lack of information about functional elements [20, 21]. Despite this trend, there are lncRNAs with high and widespread expression (MALAT1, NEAT1, TINCR) and cross-species conservation (MALAT1, TINCR, PVT1). It suggests that some lncRNAs may play a role in essential cellular processes. Thus, the study of new lncRNA genes is intriguing and requires in the experimental validation of transcript structure.

Recent studies revealed that some lncRNAs have small open reading frames (smORFs) (< 100 amino acids), that are translated. The resulting short peptides are functional and play a role in cell physiology [22]. At the same time, some studies have shown the dual function of RNAs: coding and intrinsic RNA [23].

In the present study, we investigated widely expressed lncRNA* LINC01420*, the function of which was not described at the time of the beginning of the study. We determined the structure of *LINC01420* transcript, its localization, and influence on cell physiology. During our work, D’Lima et al. found smORF in *LINC01420*. This smORF produces microprotein named non-annotated P-body dissociating polypeptide (NoBody) [24]. Authors demonstrated the potential functionality of this microprotein as a component of the mRNA decapping complex. However, our results provide new facts about *LINC01420* transcript and its function.

## Results

### *LINC01420* has conservative sequences and high expression level in human tissues and cell lines

Using nucleotide BLAST search, we revealed that *LINC01420* transcript has homologs across Mammals, but not in other Vertebrates. Moreover, multiple alignment of 100 Vertebrates genomes presented in UCSC browser confirms that this gene is present only in Mammals. HMMER analysis of NoBody homologous proteins showed the same result.

**Fig. 1:**
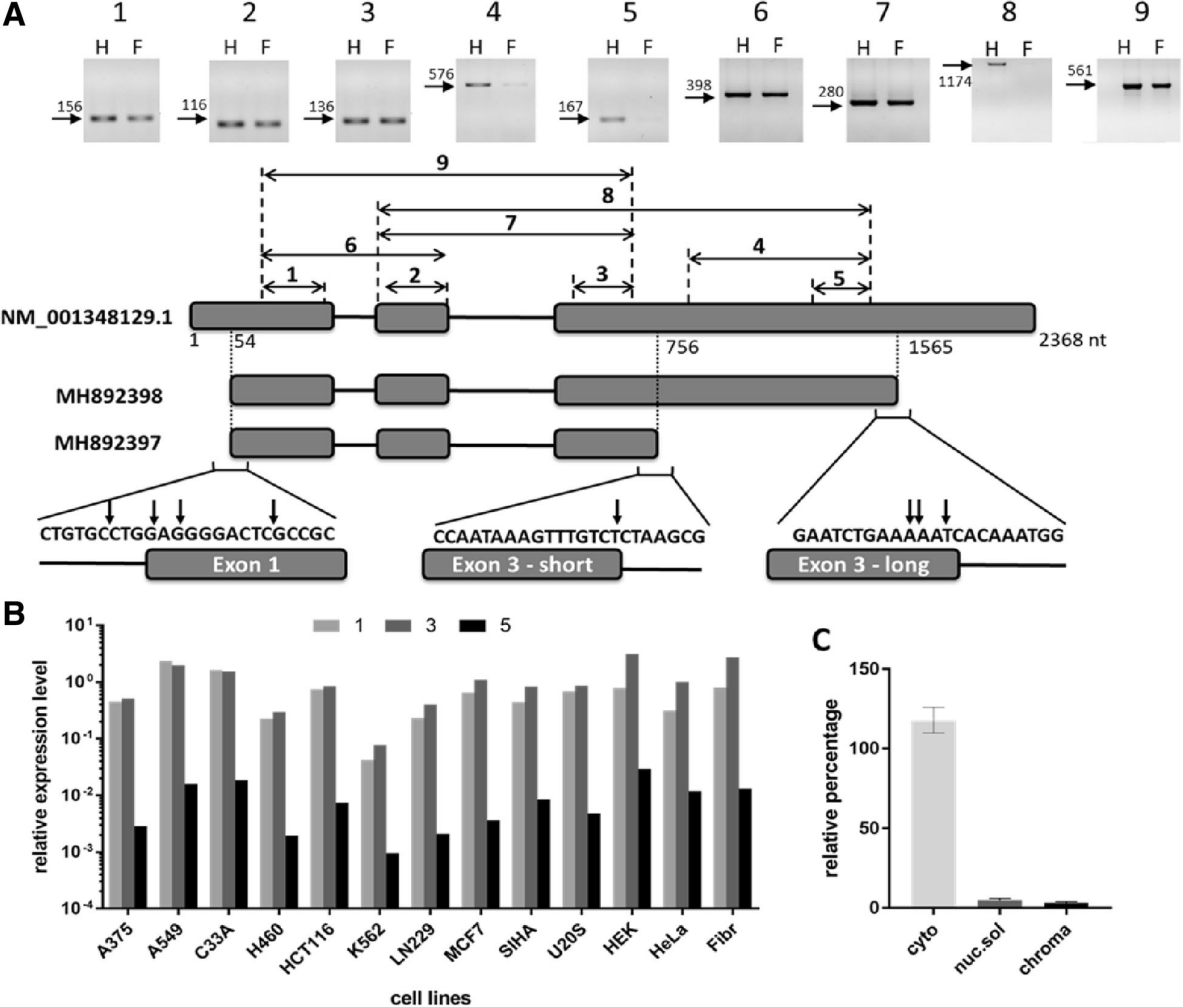
Analysis of the structure and expression of the *LINC01420* transcript. **a**. RT-PCR analysis of nine *LINC01420* loci of total RNA isolated from HeLa cells (H) and human skin fibroblasts (F) *above*. The bold numbers (1–9) indicate the amplified locus. The numbers above horizontal arrows show the length of observed products in base pairs (bp). Scheme of the ResSeq (NM_001348129.1) and two experimentally established (MH892397, MH892398) isoforms of the *LINC01420* transcript with the amplified loci are shown *below.* Results of 5′- and 3′- RACE analysis is presented *under the scheme*. The vertical arrows represent the genomic position of exact 5′- and 3′-ends. Nucleotide numbering was based on reference sequence NM_001348129.1 **b**. RT-qPCR analysis revealed expression level of three *LINC01420* loci relative to four reference genes (*HPRT1*, *B2M*, *TFRC*, *TBP*) in 12 human cell lines and human skin fibroblasts. Loci 1 and 3 are common for short and long isoforms, whereas locus 5 is long isoform specific. For illustrative purposes expression data are given in logarithmic coordinates. **c**. To identified the subcellular localization of the *LINC01420* transcript total RNA was extracted from three separated cell fractions: cytoplasmic (cyto), nuclear-soluble (nuc.sol) and chromatin-bound (chroma) of HEK293T cells. The amount of transcript in the different fractions relative to whole-cell RNA was measured by RT-qPCR. The error bars represent SEM (standard error mean)

Analysis of the FANTOM5 and GTEx expression data revealed that *LINC01420* is highly expressed in most human cell lines and tissues. Moreover, an expression profile of *LINC01420* in 975 human samples from FANTOM5 allows classifying this gene as a housekeeping gene with broad and uniform expression [25]. We validated the high widespread expression of this transcript using RT-qPCR analysis of 12 human cell lines, as well as human primary skin fibroblasts (Fig. 1b).

### *LINC01420* has two isoforms

Different lnсRNA annotations revealed various possible structures of the *LINC01420* transcript. To determine a real structure of the LINC01420 isoforms, we performed reverse transcription PCR and rapid amplification of cDNA ends (RACE) analysis on HEK293T, HeLa cell lines, and human primary skin fibroblasts (Fig. 1a). We revealed that the *LINC01420* RNA consists of three exons and has two polyadenylated isoforms that differ in length of the last exon. The total length of the short and long *LINC01420* isoforms is 701 bp and 1510 bp respectively. Nucleotide sequences of short and long isoforms were deposited into GenBank under accession numbers MH892397 and MH892398, respectively. To determine the expression level of two different isoforms, we performed qPCR with primers common for both isoforms (pairs 1 and 3) and with primers specific for the long isoform (pair 5) (Fig. 1b). We found that expression of the long isoform is ~ 150 times lower than the short one.

### Cytoplasmic localization of *LINC01420*

Since a large group of lncRNA functions is associated with the regulation of transcription and binding to chromatin, we investigated the subcellular localization of *LINC01420* using soft lysis method. RNA was isolated from cytoplasmic, nuclear and chromatin-bound fractions of HEK293T cells. RT-qPCRs were performed to determine the level of the transcript of interest in each fraction. As control transcripts, we used U1 [25] and BIRC5 [26], which are localized predominantly in the nucleus and the cytoplasm, respectively. We revealed the *LINC01420* transcript has mostly cytoplasmic localization (Fig. 1c). It suggests that the function of this transcript is not related to the transcription regulation and chromatin binding.

### The influence of *LINC01420* on cell physiology

To determine the potential effect of *LINC01420* on cell physiology we performed knockdown experiments using RNA-interference. We designed *LINC01420* siRNA and optimized transfection conditions of human melanoma cell line A375. The expression level of a gene of interest was measured by qPCR. The *LINC01420* knockdown efficiency was ~ 60% (data not shown).

**Fig. 2:**
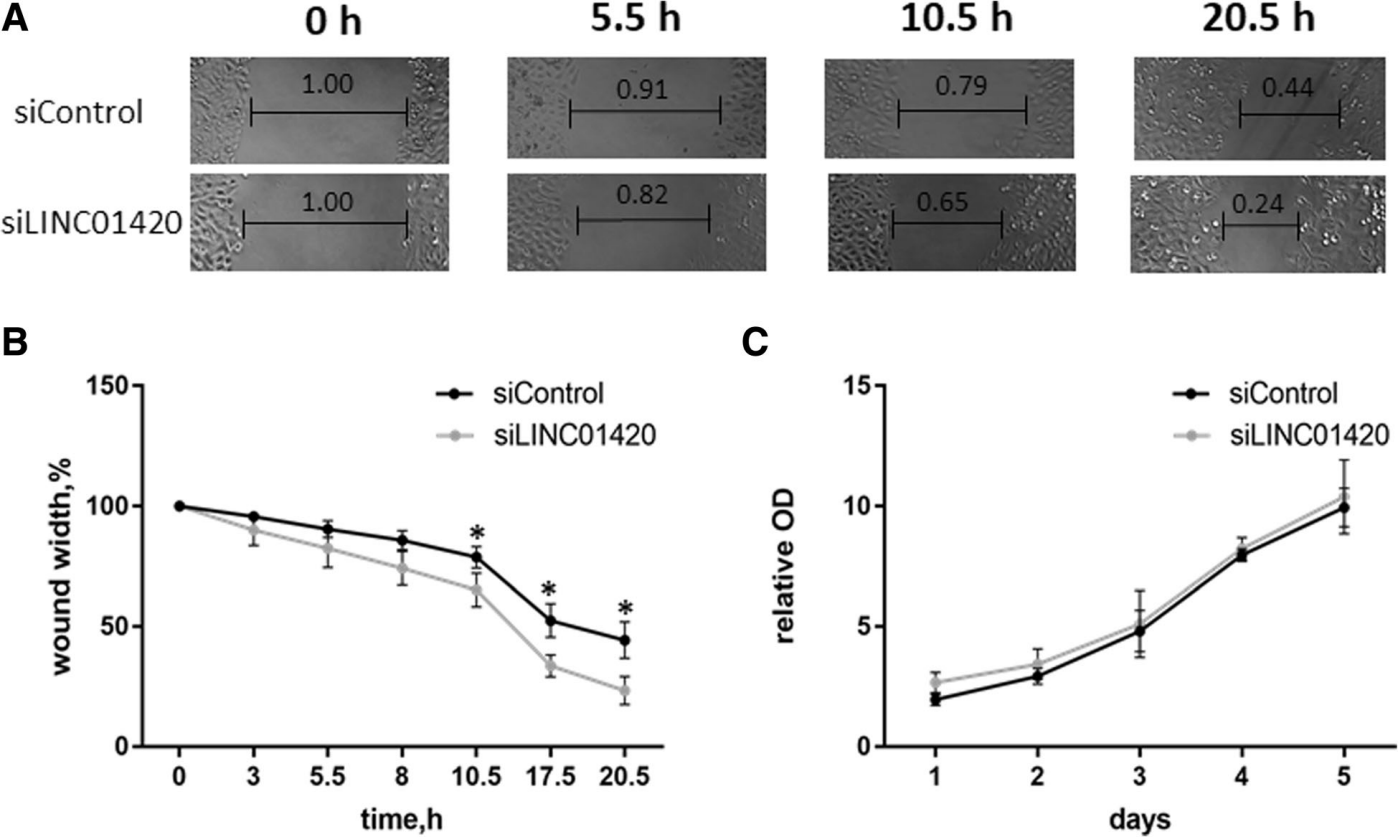
*LINC01420* knockdown revealed activation of A375 cells migration, but does not influence on cell viability. Wound-healing and MTT assays were performed on A375 melanoma cell line. Cells were transfected by siRNAs against *LINC01420* (siLINC01420) or nonspecific siRNA (siControl). **a**. Representative images of wound-healing experiments are shown. **b**. The graph shows the dependence of wound width on time. Cells treated by siLINC01420 more quickly heal the wound than control cells. **c**. Result of MTT did not reveal the difference between cells treated by siLINC01420 and control cells. Error bars represent the mean ± SEM (standard error mean) of three independent experiments. *p < 0.01, vs. control (according to Mann–Whitney U test)

After *LINC01420* knockdown, the proliferation of A375 cells was measured by the MTT assay, and the cell migration was examined by the wound healing assay. We found that the knockdown of *LINC01420* does not affect A375 proliferation (Fig. 2c), while the positive control knockdown of the *EIF3D* gene demonstrated an adverse effect on cell viability [27–29]. Wound-healing assay revealed that *LINC01420* knockdown leads to increased cell migration (Fig. 2a, b). Thus, we showed that expression of *LINC01420* inhibited A375 migration.

## Discussion

In the present study, we experimentally determined the structure of *LINC01420* transcript and revealed that it has two polyadenylated isoforms that differ in length of the last exon. We showed that the short isoform expressed at a much higher level than the long form and is localized predominantly in the cytoplasm. Besides, we found that *LINC01420* inhibited cell migration in human melanoma cell line A375, but does not influence cell viability. Recently Yang L et al. have shown that expression of the *LINC01420* gene in nasopharyngeal carcinoma (NPC) is higher than in normal nasopharyngeal epithelial tissues and correlates with NPC distant metastasis and poor prognosis [30]. The authors observed that knockdown of *LINC01420* inhibited NPC cell migration and invasion in 5-8F cell line. These contradictory results allow us to assume that this transcript affects cell migration in cell context-specific manner.

In silico analysis of CLIP-seq data about miRNA-mRNA interactions [31–34] from starBase v2.0 database [35] revealed that the *LINC01420* transcript interacts with several miRNAs. Top miRNA interactors are involved in the cell migration regulation pathways (miR-876-5p [36], miR-197-3p [37], miR-410-3p [38, 39], miR-340-5p [40], miR-873-5p [41, 42]). Moreover, expression of this miRNAs could either activate or inhibit cell migration depending on cell context. These observations confirm the contradictory results obtained in the present work and Yang L et al. However, this question requires further investigation.

Cytoplasmic localization of the *LINC01420* transcript is consistent with the work of D’Lima et al. in which the authors revealed smORF that is translated from the first exon of *LINC01420* [24]. This smORF produces microprotein NoBody (non-annotated P-body dissociating polypeptide). D’Lima et al. demonstrated that NoBody interacts with mRNA decapping protein EDC4 and loss of NoBody causes a decrease in the cellular levels of an NMD substrate. The influence of this microprotein on cell migration was not studied. Thus, it remains unclear whether the *LINC01420* effect on cell migration is associated with the microprotein or with the RNA itself.

Using SPLASH data [43] available in starBase v2.0 [35] we found that the *LINC01420* transcript interacts with *ACAD11, NPHP3* and *PPP1R3F* RNAs. However, the possible functional role of these interactions requires further investigation. At the same time, analysis of *LINC01420* RNA-binding proteins revealed that many of them are involved in regulation of mRNA nuclear export, translation and stability (e.g. EIF4A3 [44], IGF2BP1 [45], IGF2BP2 [46], IGF2BP3 [47], UPF1 [48]). It indicates the protein coding function of this transcript.

LncRNA genes became a breakthrough in our understanding of gene regulation and RNA metabolism. smORFs are a new class of genetic elements that expand the coding potential of the genome. High-throughput RNA sequencing (RNA-seq) and ribosome footprinting followed by sequencing (Ribo-seq) increase evidence of the existence of lncRNAs with smORFs [49]. Some of these microproteins are evolutionary conserved and play a functional role not related to the host lncRNA. Taking into account the common features of lncRNAs (weak conservation, low and tissue-specific expression) we speculate that most conservative and widely-expressed lncRNA genes contain smORF, as in the case of the *NBDY/LINC01420* gene. However, the question of whether microprotein or RNA itself or both determine the function of such genes remains open.

## Conclusions

We provide an analysis of widely expressed lncRNA *LINC01420*, the function of which was not described at the time of the beginning of the study. Bioinformatics analysis showed that the *LINC01420* gene has conservation across mammalian and “housekeeping” broad and uniform expression. Our experimental work revealed that *LINC01420* has two isoforms, that are localized predominantly in the cytoplasm. Besides, cell physiology experiments showed that *LINC01420* does not affect cell viability, but inhibits melanoma cell line migration.

Recently published work of D’Lima et al. revealed functional NoBody microprotein, that is translated from the first exon of *LINC01420.* However, the influence of this microprotein on cell migration was not studied. Thus, it remains unclear whether *LINC01420* effect on cell migration is associated with the microprotein or with the RNA itself.

## Methods

### Bioinformatic methods

Nucleotide sequences of the studied gene were found in following databases: RefSeq release 90 [50], Ensemble release 93 [51], GENCODE release 28. Conservation and expression level in various human cell lines and tissues were analyzed using data from the UCSC genomic browser [52]. Nucleotide sequences were analyzed using the BLAST (Basic Local Alignment Search Tool) search of the NCBI NR nucleotide database with standard parameters [53].

### RNA extraction and reverse transcription-quantitative PCR (RT-qPCR)

Total RNA from cell lines was extracted using ExtractRNA reagent (Evrogen, Russia) according to the manufacturer’s instruction. RNA was treated with DNAseI (Thermo Fisher Scientific, USA) and reverse transcribed using ImProm-II™ Reverse Transcription System (Promega, USA). qPCR experiments were performed using EvaGreen® Dye (Biotium). Primers used for amplification of different *LINC01420* loci are presented in Table 1. PCR amplification reactions were run in triplicates for each cDNA sample. For normalization we used expression of four reference genes (*B2M*, *HPRT*, *TFRC*, *TBP*), primers are listed in Table 1.

**Table 1:** Primer sequences used for amplification of different *LINC01420* loci and reference genes and siRNA sequences used for knockdown

Primer	Sequence
F1	5′ - GCCCACCGGAGAAAACTGAC – 3
R1	5′ – CCTTCCGGATAATCCCAACG - 3‘
R1–1	5′ – GAGGCTTGGCTTCCCGTG - 3‘
R1–2	5′ – TTCCAGGTGGGAGAGTGGA – 3
F2	5′ – AGTGATTGCAGTATGACTCCA - 3‘
R2	5′ – TTCCAGGTTCAGGACACCAGA - 3‘
F3	5′ – TCAGCGCGATTTCACTTCCTG – 3’
F3–1	5′ – CTGAATTTCGATGAATTCTAAGAC – 3’
F3–2	5′ – ACCTCTGAGATTTAAGGCCATG – 3’
R3	5′ – GACCATCTCACAGGCATTGTT – 3’
F4	5′ – GTACACTTTCTTTAATTTGCTGTC – 3’
F5	5′ – GAAAATGTCAGATAAACTTGGCT – 3’
R5	5′ – CTTTAATTCTGATGCTAGGGACT – 3’
HPRTf	5′ – TGTAATGACCAGTCAACAGGG - 3’
HPRTr	5′ – TGCGACCTTGACCATCTTTG - 3’
B2Mf	5′ – TCTTTCAGCAAGGACTGGTC - 3’
B2Mr	5′ – GGCATCTTCAAACCTCCATG - 3’
TBPf	5′ – CGGAGAGTTCTGGGATTGTAC - 3’
TBPr	5′ – GTGGTTCGTGGCTCTCTTATC - 3’
TFRCf	5′ – TCCTTGCATATTCTGGAATCCC - 3’
TFRCr	5′ – ATCACGAACTGACCAGCG - 3’
siLINC01420	5′-UCCGGAGAAGUAGAGAAAUdTdT-3′/ 5′-AUUUCUCUACUUCUCCGGAdTdT-3′
siEIF3D#1	5′-GCGUCAUUGACAUCUGCAUdTdT-3′/ 5′-AUGCAGAUGUCAAUGACGCdTdT-3′
siEIF3D#2	5′-CGACAUGGAUAAGAAUGAAdTdT-3′/ 5′-TTCATTCTTATCCATGTCGdTdT-3’
siControl	5′-AGGUAGUGUAAUCGCCUUGdTdT-3′/ 5′-CAAGGCGAUUACACUACCUdTdT-3′
FAM-control	5′-FAM-AGGUCGAACUACGGGUCAAdTdT-3′/ 5′-FAM- UUGACCCGUAGUUCGACCUdTdT-3′

### RACE

We isolated total RNA from HEK293T, HeLa cell lines and human primary skin fibroblasts using ExtractRNA reagent (Evrogen, Russia) according to the manufacturer’s instruction. cDNAs synthesis and rapid amplification of cDNA ends were performed using Mint RACE cDNA amplification set (Evrogen, Russia) according to the manufacturer’s instruction. All primers used for RACE are presented in Table 1. Amplicons were analyzed by 1% agarose gel electrophoresis. 5′- and 3′-RACE fragments were cloned into pGEM-T Easy vector. Ten random insert clones were obtained and sequenced.

### Subcellular localization (fractionation of RNA)

For RNA fractionation was used soft lysis procedure [12]. The HEK293T cells were detached by treating with 1× Trypsin, transferred into 1.5 ml tube and centrifuged at RT 168 g for 5′. The pellet was lysed with 175 μl/10^6^ cells of cold RLN1 solution (50 mM Tris HCl pH 8, 140 mM NaCl, 1.5 mM MgCl_2_, 0.5% NP-40, RNasin Plus RNase Inhibitor, Promega) and incubated 5′ in ice. Next, the suspension was centrifuged at 4 °C 300 g for 2′ and the supernatant, corresponding to the cytoplasmic fraction, was transferred into a new tube and stored in ice. The pellet containing nuclei was extracted with 175 μl/10^6^ cells of cold RLN2 solution (50 mM Tris HCl pH 8, 500 mM NaCl, 1.5 mM MgCl_2_, 0.5% NP-40, RNasin Plus RNase Inhibitor, Promega) and 5′ incubated in ice. The suspension was centrifuged at 4 °C 16360 g for 2′ and the supernatant, corresponding to the nuclear-soluble fraction, was transferred into a new tube and stored in ice. The remaining pellet corresponds to the chromatin-associated fraction. The ratio of fractions to total RNA was estimated using RT-qPCR. All experiments were performed in triplicate.

### RNA interference

For *LINC01420* knockdown we designed two siRNAs using in-house software.

The A375 cells were maintained in DMEM (PanEco, Russia) with 10% fetal bovine serum (Biosera, France) in a with 5% CO_2_ at 37 °C. Knockdown experiments were conducted as described in Vyakhireva et al. [54]. Briefly, 5 × 10^3^ cells were seed in 96-well plates overnight and transfected with siRNA using METAFECTENE® (Biontex, Germany) according to the manufacturer’s instructions.

For *LINC01420* knockdown we used siLINC01420; for *EIF3D* knockdown – mix of two siRNAs siEIF3D#1 and siEIF3D#2; for control we used nonspecific siControl; for transfection efficiency control we used FAM-labeled nonspecific siRNA (Table 1).

### MTT assay

At 0, 24, 48, 72 h, 96 h and 120 h after transfection of A375 cells in 96-well plate, the 20 μl of MTT (Sigma-Aldrich, USA) solution (5 mg/ml) was added to each well with 200 μl culture media and incubated 3 h at 37 °C. After this media was removed. Formazan pellets in each well were dissolved in 200 μl DMSO, and the absorbance of formazan solutions was measured at 570 nm and 670 nm (for background signals).

All the experiments were carried out at three biological and five technical replicates. Data analyzed by a nonparametric paired Mann-Whitney U Test.

### Wound healing assay

At 24 h after transfection of A375 cells in 96-well plate. Cells in monolayer culture were scraped using the pipette tips. At 0, 3, 5.5, 8, 10.5, 17.5 and 20.5 h after wounding, 1 field/well was visualized by microscopy. Then, images were analyzed using Image J program (National Institutes of Health). Changes of the remaining wound area were measured relative to total wound square at 0 h.

All the experiments were carried out at three biological and five technical replicates. Data analyzed by a nonparametric paired Mann-Whitney U Test.

## Abbreviations

DMSO: Dimethyl sulfoxide
lncRNA: Long noncoding RNA
NPC: Nasopharyngeal carcinoma
ORF: Open reading frame
PCR: Polymerase chain reaction
RACE: Rapid amplification of cDNA ends
RT-PCR: Reverse transcription PCR
siRNA: Small interfering RNA
smORF: Small open reading frame
